# Testing a novel theoretical framework to study language services implementation in health care

**DOI:** 10.1016/j.pecinn.2025.100427

**Published:** 2025-08-27

**Authors:** Allison Squires, Lauren Gerchow, Chenjuan Ma, Eva Liang, Sarah Miner

**Affiliations:** a Rory Meyers College of Nursing, New York University, 433 First Avenue, New York, NY, USA; b Abt Associates, Rockville, MD, USA

**Keywords:** Language barrier, Hospitals, Home health care, Nurses, Theory, Limited English proficiency, Medical interpreters, Interpreter services

## Abstract

Language services are used to bridge language barriers during healthcare encounters, with the goal of reducing health outcome inequities; however, the implementation of language services in healthcare is understudied. Language Planning Theory has the potential to offer a theoretical framework for studying language services implementation challenges and successes in healthcare. The purpose of this study was to test the three-level view model (3LVM) of Language Planning Theory for studying the implementation of language access services in healthcare. A qualitative secondary analysis of data generated from a study of patients with limited English proficiency receiving home healthcare services and clinicians working for the agency structured this study. Data were analyzed according to the 3LVM using directed content analysis. Results from the analysis provided insights into the factors that generate the need for language services and those that facilitate or hinder their implementation, with the theoretical framework offering clear distinctions. Analyses generated an adapted, healthcare-specific version of the model that includes clinician/staff and patient functions, which proved useful for structuring research about language access services implementation in health care.

## Highlights

• Theories specific to the implementation of language access services are few.
• Patient factors that may drive LEP patient outcomes were identified using the theory.
• Clinician and system factors that may influence LEP patient outcomes were identified.
• Findings produced a new theory for studying language access services in healthcare.

## 1 Introduction

Historic levels of global migration and growing numbers of refugees and asylum seekers have increased the likelihood that health systems may face challenges when delivering health services to people who do not speak the language of the destination country. Language barriers in health care negatively impact patient experience, quality of care, and health outcomes.[[1], [2], [3]] To improve these outcomes, healthcare professionals are encouraged to use language services like medical interpreters. [[4], [5], [6]] Research, however, highlights inconsistencies with language services (LS) implementation from the patient and clinician perspectives [4,[7], [8], [9], [10], [11]].

An emerging area of health services research focuses on studying the challenges of implementing LAS and addressing barriers at the patient, healthcare professional, health system, and policy levels. Common known issues with LAS implementation include in-person interpreter scheduling, availability of interpreters in the appropriate language, problems with telephone and video interpreter technologies, documentation of language service use in the electronic health record, and clinicians lack of trust in the translation fidelity of the interpreter [10,[12], [13], [14], [15], [16], [17], [18], [19], [20], [21]]. Nonetheless, more research is needed to better understand how to address these implementation challenges and optimize healthcare workers' utilization of LAS across healthcare settings and languages. While multiple theories exist about language development in individuals, few conceptual or theoretical frameworks are capable of systematically studying these specific challenges in healthcare.

### 1.1 Language planning theory

Language Planning Theory (LPT) has the potential to guide research on the implementation of language services and their resulting outcomes.[22] LPT was developed in the mid-twentieth century as a subfield of political science to plan for multi-language communication and its role in shaping political dynamics at the country level.[23] The theory posits that the identification of language problems at the interactional level leads to the adoption of language planning efforts at the institutional level to facilitate the function of LAS [22]. In healthcare, this would occur between providers and patients, resulting in organizational planning to facilitate it.

Importantly, the tenets of LPT consider the significance of the implementation context and the possibility that one language may be more prominent, known as “language dominance.” For example, English is the dominant language in the US. In healthcare, failing to act and bridge a language barrier through the provision of language services results in inequities between speakers of the dominant language and those of the non-dominant language. Implementing language planning, however, lacks conceptual and theoretical guidance specific to healthcare settings.

**Fig. 1:**
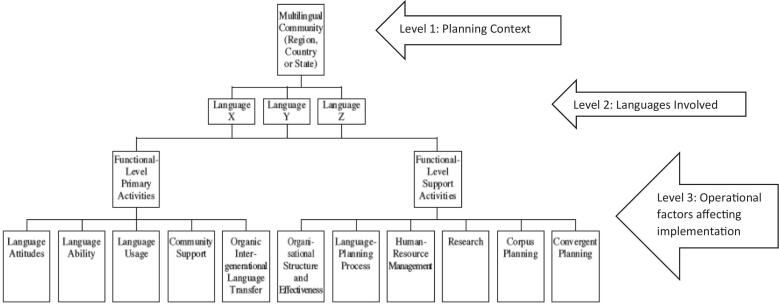
MacDonnacha's three level view of language planning [24].

The purpose of this study, therefore, is to evaluate the applicability of Mac Donnacha's Three Level View Model (3LVM) version of LPT (Fig. 1) as a theoretical framework to study language service implementation in healthcare.[25,26] Mac Donnacha employed strategic business planning principles and harmonized several language planning models to enhance planning efforts that consider the implementation context. We propose that the organization, provider, and patient must all be considered simultaneously to fully comprehend the successes, limitations, and failures of language planning efforts in health care, which the 3LVM model captures.

## 2 Methods

### 2.1 Design

We conducted a qualitative secondary data analysis of interview data collected as part of a large study examining how language barriers affect the delivery and outcomes of home health care (HHC) services. Qualitative secondary data analyses use existing qualitative data to answer new questions.[[25], [26], [27]] Institutional review board (IRB) approval took place at the lead author's home institution (IRB# 15–10,710) and the participating agency's (IRB#: E15–008).

### 2.2 Setting

A HHC agency in the United States (US) served as the contextual test case for the study. In the US, HHC services include home-based care from skilled professionals, such as nurses or physical, occupational, and speech therapists, for individuals who are homebound and unable to care for themselves independently. Furthermore, federal regulations stipulate that access to language services is a civil right, and individuals with language barriers must be provided with resources to communicate in their preferred language in healthcare settings.[28] HHC services are an understudied context for the implementation of language services; [10] thus, it serves as a practical case example for testing the theory through the proposed analysis.

### 2.3 Description of the parent study

Conducted between 2015 and 2018, the parent study was conducted in partnership with a large HHC agency on the East Coast of the US, which serves a significant patient population with limited English proficiency (LEP). A person with LEP, which includes deaf persons who use sign language, cannot communicate safely in English and requires an interpreter to be present to facilitate communication.[28,29] Both qualitative and quantitative data were collected to examine how language barriers affect service provision, patient experience, and 30-day readmission rates.

### 2.4 Population studied

Multiple stakeholders were included in the study. Patients (majority over age 65 and 58 % female, insured by Medicare) had received home health services in the last year and self-identified a preference for communicating in the top languages served by the home health agency: Spanish, Chinese (i.e., Mandarin and Cantonese dialects), Korean, and Russian. Patient volume served by the healthcare organization fulfills Level 2 of the 3LVM model, “Languages Involved”. Participants also included registered nurses, physical and occupational therapists, nurse managers, and nurse care coordinators. Eligible professional participants had worked in HHC for at least one year and averaged 21 years of healthcare experience (range: 6 to 32 years).

Recruitment and data collection processes were tailored to role (patient versus provider; provider type). Patients' semi-structured interviews occurred in the home or via telephone with a language-concordant research assistant (RA). Provider interviews occurred by phone or in-person with AS or SM. Informed consent was obtained at the beginning of each interview. All participants received a $30 gift card as an incentive. All interviews were digitally recorded and lasted between 25 and 100 minutes. Transcription varied by language. Detailed descriptions of the methods of transcription, translation, and quality verification for both populations are available in separate publications.[30,31].

The final sample size comprised a total of 70 patients (18 Spanish, 15 Mandarin, 12 Cantonese, 12 Korean, 13 Russian) and 34 providers (30 registered nurses, three physical therapists, one occupational therapist; 14 in a management role; 12 spoke another language besides English fluently). Spanish-speaking patients were primarily of Puerto Rican and Dominican heritage. The number of years lived in the US in the patient cohort ranged from seven to 30 years.

### 2.5 Data analysis

To conduct the qualitative secondary analysis, we used directed content analysis guided by the 3LVM model (See Fig. 1). In the model, “primary activities” generate the creation of a service. “Support activities” focus on reinforcing communication in the language and thus reflect implementation-related actions by key actors. The definitions of all concepts in 3LVM met our analytical needs without requiring adaptations for healthcare, except for the concept of “Corpus Planning.” This concept traditionally focuses on creating written translation resources, such as dictionaries, to facilitate effective communication. Through consensus, we agreed that for healthcare, the equivalent concept would be the availability of relevant documents, such as patient education materials, in the patient's language. Furthermore, while the categories from the theoretical framework guided the analytic process, the researchers remained open to new codes that were not salient with the initial categories, as recommended by Hsieh and Shannon.[32].

AS and SM coded the data. Both are bilingual nurses, health services researchers, and cisgender women of European heritage, one with extensive experience in HHC. Throughout the coding process, they intentionally situated their perspectives within the context of the first two levels of the 3LVM (a HHC organization in a multilingual, urban setting). After coding four interviews, they performed dependability checks conducted through random review of the others' codes, allowing for early codebook harmonization. Once finalized, they presented the final themes and categories to the research team as a confirmability check.

## 3 Results

The setting of the parent study and the qualitative data, in effect, encompass Levels 1 and 2 of the 3LVM model, thereby creating the analytic context. The presentation of the findings is guided by Level 3 of the 3LVM model framework. Patient codes primarily fell under the “Functional Level Primary Activities.” Provider-centered findings fell under the category of “Support Activities.” We present the findings in two thematic groups aligned with Level 3 of the framework, renamed according to our analyses: 1) patient-centered functional level primary activities; and 2) organizational and provider-driven functional level support activities.

### 3.1 Patient-centered functional level primary activities

The need for language services emerged based on the patient-centered primary activities in the model. Patients described consistent experiences accessing and using language services during healthcare encounters, regardless of their language group. Table 1 provides the supporting quotes for the patient-centered functional activities section of the theoretical framework. The succeeding sections review the details of the analyses.

**Table 1:** Supporting quotes aligned with patient centered functional level primary activities.

	Supporting Quotes
Language Attitudes	
Cantonese	Like mainly I….yes…mainly because we are Chinese so those are your own problems. I mean America's primary language is English. You can't ask for too much, it's just that you need to speak through an interpreter [to understand]. Like that.
Korean	It was little frustrating. There were professional jargons and not every day conversational language that I use. And since English is my 2nd mother language, there are parts that I cannot understand and had some discomforts.
Mandarin	Kind of like, you don't speak English, now, for example you're Chinese, you obviously wish there's Chinese people, or, you're Mexican, you wish there's Mexican people, right? This is just my own wishes, hope, but you have to think about the situation. Because there's still the other country's people, right? You cannot expect the hospital to be full of Chinese, correct, am I right?
Russian	The Russian language, only Russian. No, I do not know (English). I used to know. I forgot what I used to know. (The patient laughs) Yes, when I was taking my citizenship test, I studied, studied everything by heart and passed. And later I forgot everything.
Spanish	Yes it affects me, because I wanted to speak English, because I have lived here for so many years. But when I arrived here and my children were in Honduras, I didn't have time to go to school. So I didn't. I hear the English spoken on the streets, not the English from school. Because it's different English in the streets than in school.

Language Ability	
Cantonese	I don't understand [English]. A little bit of it, like having a surgery, *operation,* these I know. Because during that time, I stayed at the hospital. But whatever appendicitis is, I don't understand; that's why I feel kind of embarrassed. If they have this interpreter, I think it will not be a problem for a few hours.
Korean	Yes, and at first because I am not an English speaking person, I tell them in advance that my English is not enough. So speak slowly. And if there are any medical words that I do not understand, give me the spelling. Then when you put it in, it comes out.
Mandarin	Because after all I'm Chinese, as a Chinese, I can speak simple English, like if I'm socializing with others, I can speak it, but if I encountered something more difficult like technical words for instance, I don't really know, I couldn't communicate with those.
Russian	Like my language level is enough for me to understand everything. If some nuances, um, occurred when I did not understand something, I tried to look up something on my own there. Some translations or something like that…Well, I tried. If in this, um, in that, in that office or some other place, there weren't any Russian speaking people. If they were there, then I tried to ask them something. If not, then I tried to find out on my own the things that I did not quite understand.
Spanish	I can't listen in English [to medical conversation] because even though I understand a little bit I don't know how to communicate in English. So even though I understand, I won't be sure when listening to medical things. Hearing about medical things makes me very afraid, you have to be very sure.

Language Usage	
Cantonese	There was a time when I was taking the medicine and I don't know how to tell them where the pain was or they need to explain to me which medicine to take then there was an interpreter. When I couldn't explain it clearly then I needed an interpreter.
Korean	When I went to this hospital, even though I cannot speak English perfectly, they make it so that I can understand. With hand gestures or they hold this and say something, then I can understand that.
Mandarin	Because you see there's a lot of elderly, or, others who don't speak a word of English. Even for me, I know simple English I still need an interpreter. ‘I need a translator. I speak Mandarin’. There's a lot of elderly, they don't even know how to say these simple sentences. So I think it's a hassle, it will take up time.
Russian	There, it turns out a little bit… I told you already that I have been here, in America, for a long time. I speak English. And when I am letting…um, the fellow knows right away, um, that I have problems with English…and clearly. Then, u-um, when I ask one more time, u-um, and I use a pencil and a sheet of paper. We understand each other perfectly. If the person is enthusiastic to communicate with me.
Spanish	I would prefer [to communicate] in English. Do you know why? Because many doctors say exactly what they mean to say when they speak English. They communicate better. But if I realize I'm not understanding well then I speak Spanish.

Community Support	
Cantonese	When I came, I just went to sleep, I didn't even eat anything and went to sleep. Slept until the middle of the night, I felt some worms on my head. So I went back to Chinatown. The doctor in Chinatown said yes, it is *shingles*. And immediately prescribed me medicine, gave me…the one in Chinatown speaks Chinese…gave me those antibacterial medicines to take.
Korean	That's right. I'm glad that I met a Korean primary care provider, and when I go to different place to do something, I can't speak in English, she [my daughter] hears and she was here, so my heart….
Mandarin	Pharmacy, I look for Chinese pharmacy. (Oh, due to the ease of communicating?) Right, yes. So the doctor tell me, ‘I'll prescribe what medicine for you.’ then I go to the pharmacy and pick it up. They will also explain it to me, how to take this medicine, etc. It's all in Chinese. (Oh, so the pharmacy employee will explain it to you in Chinese, right?) Yes.
Russian	Well, being honest. I haven't had any problems. [At the local hospital], there are Russian doctors there, there are Russian nurses. A-and I-I not so. Well, u-um, I-I am n-not very good in English. But I understand when a person is next to me, I understand everything.
Spanish	At the pharmacy they speak Spanish […] For example, I call the pharmacy […] and they already know I am (patient's name) […], they start speaking Spanish. The owner and everyone else speaks Spanish.

Organic Intergenerational Language Transfer	
Cantonese	If it's the doctor…if my family is there the same time the doctor make rounds then my family will help me translate. But I think the doctor coming with an interpreter is better. Because my family do not have knowledge in the medical field. Sometimes some of the medicines they don't really understand what it says. So I think…my two sons they definitely don't understand, they did not study this field or learn this. So they obviously don't know. Unless it's my daughter in-law, but she get off work and visit me before she go home.
Korean	When I received healthcare service, I went with my son, and my son spoke English so I had no [problem]….
Mandarin	Sometimes the doctor come, there is a Chinese interpreter, sometimes they will call my daughter. My daughter will tell the doctor about my situation, so the doctor will know about it.
Russian	The most preferable interpreters for me are my children because they know specific details of my illness.
Spanish	For example if my daughter is there I just tell her that whatever they were going to tell me they should tell her. […] When I don't know and don't understand, I can't ask them any questions in return.

#### 3.1.1 Language attitudes

Language attitudes are reflected in patients' understanding, willingness, and judgments around their language use across health care settings. Regardless of language group, patients often expressed feelings of guilt and frustration for their inability to communicate in English during healthcare encounters, especially for those who had resided in the US for “long periods.” Their perceived sense of “burden” varied widely due to their lack of English competence. The majority of patients had a positive attitude about learning the dominant language.

#### 3.1.2 Language ability

Overall linguistic competence shapes language ability within the four standardized domains of speaking, listening, reading, and writing among patients. Interviews revealed literacy issues in the patients' native languages, especially among elderly Spanish, Mandarin, and Cantonese speakers—the latter of whom were more likely to speak a Chinese dialect with limited diffusion. Russian speakers reported the highest overall language abilities, with all participants demonstrating competence in the four standardized language domains. Many of them also spoke Ukrainian and Tajik.

About one-third of patients indicated that they could understand a “substantial” amount of English. Expressed English language ability was strongly tied to the decade of immigration to the US (e.g. 1970s versus 2000s.). Participants also linked their language abilities to the extent of schooling they completed in English and the overall quality of their childhood education in their country of origin.

#### 3.1.3 Language usage

The two aforementioned categories are subsequently tied to the participants' language usage, which manifested in how they described their a) use of language in health care encounters and b) their preferred interpretation modality. Patients uniformly agreed that they favored providers who spoke their preferred language. Many actively sought out these providers. Patients made decisions about language usage based on self-assessed language abilities across the four domains. They described listening comprehension as their common “usage” in healthcare encounters. They felt that this “usage” was compromised when providers used medical language extensively, even with an interpreter present.

Patients distinguished between situations where they viewed interpreter services as “critical” to their language usage and situations where they felt comfortable using body language to express their needs. They emphasized that interpreter services were essential during “critical” points in a health care encounter, such as providing informed consent, hospital admission, and discharge, events that align with the legal requirements for implementing language services. During “less critical” interactions, like toileting or ambulating, patients reported using body language, gestures, or other nonverbal cues as sufficient to communicate their needs.

#### 3.1.4 Community support

Patients tended to live in communities where people spoke their language. “Community” also meant that patients had greater access to pharmacies and health care providers who spoke their language. More than other patients, Chinese and Russian-speaking patients have access to language-concordant providers in their communities.

When receiving health care services outside their community, patients reported more problems associated with language barriers. Taishanese and Shanghainese Chinese dialect-speaking patients, in particular, reported more negative experiences than other patient groups, even in settings with high levels of language concordance in Mandarin or Cantonese. Examples included less frequent access to interpreters or longer wait times when interpreters were not coordinated in advance of an office visit to see a clinician.

#### 3.1.5 Organic intergenerational language transfer

The nature of immigrant communities meant that as long as families remained within the community, intergenerational transfer of language skills was likely to occur. Patients often reported that their children had higher levels of literacy and proficiency in the four domains of English than they did. When children moved outside their community, however, challenges emerged for patients. Older adults' children often had language skills that stagnated at the “heritage speaker” level, meaning they could speak and understand their parents' language with an everyday vocabulary. They were also less likely to read and write in their parents' language, which increased the risk of misinterpretation during healthcare encounters where they served as their parents' interpreters.

Local healthcare organizations also leveraged the organic intergenerational language transfer process. For example, children who remained in the community continued to interpret for their parents or family members during healthcare encounters, conveying information and concerns to providers. They could also easily read hospital discharge instructions provided in English or in their parents' preferred language, depending on their language abilities. Some facilities in multilingual communities had discharge paperwork available in patients' preferred languages, but limited literacy, more frequently expressed by Spanish- and Chinese-speakers, often precluded these patients form understanding.

Participants highlighted the qualities of local healthcare organizations that prioritize healthcare materials in the patient's language. These organizations typically employed providers who spoke the patients' languages to address the gaps that result from variation in familial language skills (or the absence of family altogether). While providers did not always live in the local community, their language fluency was most important to the patients. This approach earned the loyalty of patients, which they believed contributed to improved continuity of care and overall management of health conditions.

### 3.2 Functional support activities by providers and the organization to facilitate language services implementation

“Support activities” came mainly from staff interview data, though there was some overlap with concepts found in primary activities. These activities helped support efforts to implement language services, thereby reinforcing communication in the patient's preferred language where possible. Through that pattern, it met the conceptual definition of “support” from the original model.

Overall, the staff interviews reflected the complex challenges they face when implementing LAS in HHC as a support function within their organization. Staff views on LAS implementation depended mainly on their roles and self-reported language skills, and the process overall appeared more organic than strategic. Table 2 contains supporting quotes for each of the succeeding sections.

**Table 2:** Supporting quotes aligned with organizational and provider centered functional level support activities.

	Supporting Quotes
Organizational Structure and Effectiveness	
Monolingual Speakers [MRN4]	Again, what I would say would be helpful is to put back the nurse, the home care nurse, as the discharge planner. He or she is not the discharge planner anymore. It's social worker. Who better knows what the patient may need than the home care nurse, the discharge home care nurse, the home care nurse? I find that the discharge plan is very, very limited and restricted.
Other Language Speakers [BRN2]	Having the language line, which is what we use—the company we use to help with translations. They're available fairly late into the evening. I've seen patients at 6:00 p.m., and I've had that option to have a translator, which is great.
Managers [MG14]	I think having that language literature, like discharge paperwork, consent forms, is a difficult issue because the health information privacy form is very difficult concept to understand for the patients. When we request them to sign, usually they sign with very minimal understanding. Except for the first consent to services form—so you haven't any type of brochures, letters to the patient saying this, and that's written in their language—will be helpful.
Care Coordinators [CC3]	Well, a lot of them are—or I don't wanna say a lot of them, but there's often the case where there's other social issues where family is involved. The family is neglecting their care. Very easily, some, they could help out, especially if they can read English in the home. The discharge summary, more often than not, I don't think I've ever seen it in Spanish.

Language Planning Process	
Monolingual Speakers [OT1]	[Referring to working with LEP patients] That's my challenge during the day sometimes. Sometimes, some of these patients, I'm the only one beside the home health aide that they see. ‘Cause nobody come around. When they see me they want to keep me there, they talkin’ and talkin’, you know. ‘Cause then they have so many questions to ask me about the medication, how to take it. Or the insulin, or the blood sugars, you know. I go over that with them. The need a lot more time.
Other Language Speakers [BRN1]	That's one thing that we could do better. If we could at least ensure that the employees that do provide translation services for patients so patients going to a Chinese clinic—the person that spoke Chinese also spoke English well enough to deal with people like myself that doesn't speak the language.
Managers [MG1]	I think the person-to-person interpreter was great. Especially with our elderly and hard of hearing. Obviously, you can't have everyone who's not English proficient get an interpreter, but, at least, if you identify an elderly person who's hard of hearing, that might make a difference.
Care Coordinators [RNCC4]	What happens is that the patient didn't have—I believe the patient had a family member at the bedside that spoke both languages but wasn't a hundred percent fluent in English, but she did understand enough to be able to understand that they couldn't get a translator and explain that to the patient. Then I was provided with a phone number for another daughter who did speak English but didn't happen to be present, and I had to track down a family member that could speak English because I couldn't find anyone to translate.

Human Resources Management	
Monolingual Speakers [PT2]	[*Referring to how personnel are assigned to patient*s] They know because they usually have, it depends where it's coming from. If it's from the hospital base there's a person scheduling it. I don't know the exact title. Hospital Coordinator, HCC I think. They put notes in, patient da da da. Only speaks Spanish. Then that goes into the person that schedules the assignments, which everyone is here they're on the fifth floor.
Other Language Speakers [BRN7]	Even doing it on speakerphone, it's helpful. It's better than nothing, but sometimes, there's a hearing issue, whether the translator doesn't always hear everything, or the patient doesn't hear everything, especially if they're elderly, and hard of hearing. I think face-to-face tends to be better. We also have the possibility to bring an escort with us that may also function as a translator, but I tend not to do that. Then you would just have the person going out with you who could translate on an ongoing basis.
Managers [MG7]	Even though we know they're Spanish speaking, we know that they have to—but at least if they put it in [the chart] there somewhere saying, identify them, identify the patient saying, okay, they would like [a Spanish speaking nurse]—then right away, you schedule—when you look at the comments, you say, “Okay, let me put Jackie on this case,” or whatever. Let me put this special nurse, Spanish speaking nurse, on this case. Even though it might say Spanish-speaking. Let's say Jackie's off or something like that, she could probably have somebody else open the case, but you put it back to her because at least you know that there's like a wish, a request from the patient from the get-go.
Care Coordinators	Well, re-hospitalizations have been in the spotlight in the recent years connected to the Affordable Care Act, and hospitals have departments created that're called care coordination, where they're trying to prevent hospitalizations and just finding care coordinators that're language-concordant would actually help.

Corpus Planning	
Monolingual Speakers [ML3]	Does this translator speak that dialect? It's not just being a Spanish translator, do you know the dialect?
Other Language Speakers [BRN1[]	We find out that this patient is back in the emergency room and you do a little follow up, what went wrong? Was there a translator there? No. Was anybody home? No.
Managers [MG12]	There's a lot of systems that allow us to push the button to say, “Two for Spanish,” but I don't know how much beyond that they're able to get their needs known, if they're able to get through. I don't speak Spanish so if I had to go through the Spanish speaking line I wouldn't know if they're getting more help that way or not, or what their level of frustration would be. That type of thing.
Care Coordinators [RNCC6]	*Interviewer:* Why is that a problem? Why is using family a problem or not using the translator line a problem? *Interviewee:* Well, I think it's a problem because the patients and the families won't understand what the patient is supposed to be doing at home. Sometimes that can cause an issue if they're not taking their medication appropriately or if they end up readmitted because they—the situation got worse or if they're supposed to follow up with the doctor, and they don't because they think it's on a certain day, and it ends up being another day because they misunderstood. That's an issue.

Convergent Planning	
Monolingual Speakers [MRN5]	I have made a point on a number of occasions and so have other nurses the fact that we are, we're not in sync in terms of getting that information and getting the right information, the right doctors. Often times I have patients who come to me and have no community doctors. No care givers. They have no insurance information. They have really nothing, and they don't speak English.
Other Language Speakers [BRN5]	If they can't ask, they—in general, they don't understand how the healthcare system works. They don't know anything about, let's say, insurance. They don't know what they write. They don't know how the hospital works because the system is totally different. They get confused. They used to be almost all their life in some different situation and different system, and different rules for everything. When they got here, they learn quickly, but they need someone to go and explain everything to them, someone that will guide them. That's why the Russian doctors, like the community doctors, they prefer to have, let's say, visiting hours to visit him and guide them to tell them how their system works, how often they have to see doctor, how often they will have a blood test or any kind of other tests. Because they did not get used to that, and they don't know this system.
Managers [MG9]	With the staff, no, because they speak the language, and they're in front of them. They're nurses whereas the translator is not a nurse—I don't know if they're nurses or not, and they might not understand what I'm trying to get across to them, because it has to do with healthcare. That's my only concern.
Care Coordinators [RNCC5]	[*Referring to community outreach to address health issues]* We reached out to, let's see, adult day centers. It was mostly adult day centers. They were usually attached to the company that the company has a contact with the day center. Once a month, we would go in to teach about diabetes or tuberculosis or a side effect. What if you had pneumonia, what to do or how to recognize the symptoms that you're not taking your insulin properly. Again, everything goes right back to diabetes or hypertension, just teaching them that.

#### 3.2.1 Organizational structure and effectiveness

HHC agencies' specific ability to respond to patients' needs for LAS appears to depend on their internal language planning processes and those of the referring organization. Staff described these processes as either consistently working with interpreter services or using improvisational techniques to communicate with patients. Their success, however, depended heavily on how the information transfer was handled between the referring organization and the receiving agency. For example, when a hospital provided discharge instructions in the patient's language but did not have an English-language copy that home care staff could read, the HHC staff had to do extra work to determine what was on the discharge plan.

Staff perspectives on the importance of language-concordant encounters influenced views on LAS needs. Multilingual staff and managers prioritized concordant encounters, regardless of clinical need, when compared to monolingual staff, who described telephone interpretation services as sufficient to facilitate the visit. Care coordinators organizing referrals from the hospital often took steps to ensure that they visibly documented a patient's language preference, but expressed frustration when home care staff ignored that information and did not contact patients in the appropriate language. While interpreter services were consistently accessible to staff, patient education materials were often unavailable for staff to use with patients, even when facilitated by an interpreter. If materials are available, telephone interpreters do not have access to written materials specific to the organization, which limits the interpreter's effectiveness. This affected HHA staff's ability to conduct teaching with patients and family members to promote self-management. No staff participant reported that they had considered whether or not the patient had basic literacy in their preferred language to understand language-concordant educational materials.

#### 3.2.2 Language planning process

Language planning processes refer to how organizations develop policies and implement practices to bridge language barriers. Participants described organizational policies and actions aimed at complying with the law, ensuring good patient satisfaction scores, and managing costs. There was an overall preference for in-person interpreters, who were an agency resource in previous years but were no longer available after budget cuts. Advanced planning to meet the patient's communication needs was often challenging due to the hospital's inadequate communication during the home care referral process.

#### 3.2.3 Human resource management

Although at the time of the study, the participating agency conducted no formal language skills assessment and relied mainly on employee self-report, all staff had language skills captured in human resource records. In interviews, most bilingual staff reported language skills slightly above the level of heritage speakers and described good “medical language” fluency in their other language(s). Managers who spoke languages reported taking steps during new hire orientation and probationary periods to verify the self-reported language skills through patient follow-up phone calls or field observations. Verification was not official organizational policy, but actions managers deemed necessary to ensure “safe” communication practices and client satisfaction. Monolingual managers did not report taking similar steps to verify the clinician's language skills.

Managers also frequently lamented their inability to hire enough personnel who matched the demand for language-concordant providers. Spanish-speaking professionals were reported as the most difficult to recruit, even though Latinos/Hispanics had the highest demand for home care services. Russian-speaking nurses were described as easier to recruit–many of these nurses had Ukrainian heritage and spoke Russian as a second language. Many Korean-, Mandarin-, and Cantonese-speaking patients were able to receive language-concordant care if they lived in a community where their language was commonly spoken. Managers, however, reported insufficient availability of Mandarin- and Cantonese-speaking nurses to meet the demand.

Staff expressed displeasure about their organization's decision to eliminate the in-person interpreter program to cut costs. They viewed in-person interpreters as critical partners for health promotion and literacy activities, medication management, patient safety in the home, and reducing risk for hospital readmission. Due to connectivity challenges with telephone and high-speed internet service in patient homes, staff did not think it would be feasible to use video interpreters in the majority of cases, despite its potential to simulate in-person interpretation better than audio interpretation alone.

#### 3.2.4 Research, corpus planning, and convergent planning in other areas

These three aspects of the model appear interdependent in the HHC context. The case exemplar organization in this study has a positive culture toward research and includes a research division, which is atypical for most home care agencies. Participating staff reported above-average rates of translation of research into practice based on studies conducted within the organization; yet, there was no strategic research direction focused on enhancing language planning activities at the time of this study.

Data from the staff participants also suggest that partnerships to enhance the hospital-to-home care transition process would be an important strategic element in both Corpus and Convergent planning activities, due to their potential to reduce inequities in patient outcomes, including readmissions to the hospital. Staff recommended several actions to address vulnerability points in inter- and intra-organizational language planning processes: 1) Consistent documentation of the patient's preferred language in the EHR and transfer documentation in all the “correct places”; 2) discharge instructions in both the patient's language and English; 3) quality improvement follow up of reasons for a delayed first homecare visit when a language barrier is present to determine if staff or patient factors were involved; and 4) tracking rates of language concordant visits and/or the consistency of the same provider delivering care.

## 4 Discussion and conclusion

### 4.1 Discussion

Findings from this study suggest that the 3LVM version of Language Planning Theory, proposed by Mac Donnacha, can be an effective theoretical model to guide studies of LS implementation in healthcare settings, as it directs the analytic focus toward discrete factors associated with effective language planning practices. The use of the framework has the potential to inform care delivery broadly, including the effects of language concordant encounters on patient outcomes, to determine the appropriate “dose” for optimal results.

The few studies that have investigated LS implementation in health care used existing implementation science frameworks [33,34]. While these frameworks are useful, they have a broad focus that may overlook specific factors that affect LS implementation that are included in the 3LVM, such as the patient specific factors of language ability and language attitude.

The framework also helped our analysis identify and confirm the persistence of ongoing issues with LS implementation as linked to patient outcomes which are supported in the literature. These include the quality of the referral process from the hospital [[35], [36], [37]], patient-provider relationship development [[38], [39], [40], [41], [42]], and the effects of continuity of care on patient outcomes.[[43], [44], [45], [46]].

#### 4.1.1 Implications

**Fig. 2:**
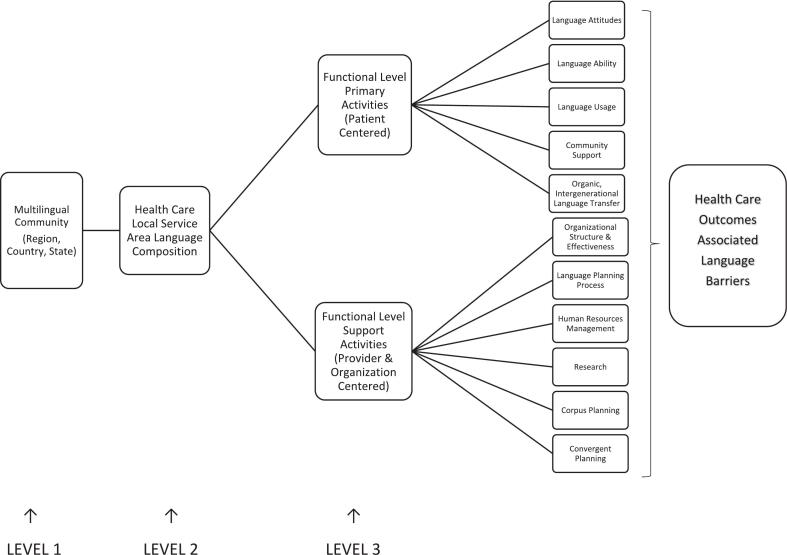
A conceptual model of the three level view of language planning for health care delivery and outcomes research.

Based on this analysis, we propose a healthcare centered adaptation of the model based on the findings of this study (Fig. 2). The organization of the model into distinct patient-centered and provider-centered structures illustrates the distinctiveness of issues experienced by both parties with language barriers during the healthcare encounter, what factors are unique to each group, and how the branches of the model may lead to specific patient- and organization- centered outcomes. Consideration of the three levels provides insight into the overlapping individual, organizational, and societal factors that may influence the implementation of language services. The adaptation can guide researchers in identifying the determinants and mechanisms that may influence outcomes, and the qualitative data provide insights into strategies to make language services more effective, feasible, and acceptable for patients and healthcare professionals. Research should use the model to differentiate its efficacy as a theoretical framework to guide assessment or evaluations of LAS implementation.

From left to right, the model begins with the multilingual community served by a local healthcare organization. According to our findings, patients who speak the same language tend to reside in the same community, which tends to grow over time. Eventually, the demand for health services in the community's language increases sufficiently that the organization must adapt beyond the legal requirements of the state or country. This is the point at which planning needs to occur, divided into two areas: Functional-level primary activities that are either patient-centered or specific to providers and organizations. Table 3 summarizes the components of the patient and provider/organization functional activities identified in the model in the healthcare context.

**Table 3:** Definitions associated with the health care centered model.

Functional Level Activity	Definition
Primary - Patient Centered	
Language attitudes	Attitudes of the community toward sustaining the language being spoken in the community.
Language ability	The classic definition of language skills as defined by a person's speaking, aural understanding, reading, and writing abilities. These may vary widely in a community and writing abilities also depend on if the language is written, which may not be the case for many indigenous languages.
Language usage	How the community actively uses the language, including if reading and writing in the language is sustained.
Community support	Behaviors in the community that sustain the active and passive use of the language within it.
Organic, intergenerational language transfer	How the community sustains the use of the language across generations. This may include a preference to use family members as interpreters.

Support – Provider & Organization Centered	
Organizational structure & effectiveness	How the health care organization implements language access services in response to demands from the community and legal requirements. This includes if laws that guide language access services implementation are enforced.
Language planning process	How organizations plan for, evaluate, and determine the cost effectiveness of language access services implementation in health care. It may follow any kind of strategic organizational planning approach, but the intentions should be linked to standardized patient outcome measures to gauge the quality of the planning process.
Human resources management	The process by which the organization will respond to demand for language access services in health care through the hiring of interpreter services–be they in-person, telephone, or video—and language concordant personnel. It includes limits placed on the use of non-interpreter certified health care team members to interpret between patients and providers as well as computer-based translation services that are not verified for their consistent accuracy when used for interpretation in health care.
Research	The work completed as part of language planning processes and eventual implementation. This includes both drawing from the evidence-base, on-going evaluation through quality improvement initiatives, and when possible, conducting formal research studies.
Corpus planning	Corpus planning facilitates access to materials in the language of the patient as needed to implement services provided by clinicians. This action reinforces the patient's ability to use their preferred language during the healthcare encounter.
Convergent planning	Whilst normally conducted a national level, in the case of health care this area would be how the organization responds to laws or policies that establish minimum requirements for how to respond to demand for language access services even when demand may be a lone individual or a small community that speaks a language of limited diffusion.

Importantly, the only aspect of the model that did not clearly appear in the data was the provider-centered branch focused on research. Despite the partner agency having a research division, the translation of “research” into everyday practice was not apparent in the data. That is likely more the result of the questions asked of the participants since the agency itself has a strong evidence-based practice approach to care delivery. Thus, changing that aspect of the model to “Research and Evidence-based Practice” may better reflect actual front-line operational experiences of health workers and organizational planning.

Future research may help determine if the variables identified in the model have causal, predictive, mediation, or moderation effects on health outcomes. The type of organization will likely affect these relationships and thus, testing the theory should rigorously account for context.

#### 4.1.2 Limitations

Methodological limitations of qualitative secondary analyses include the inability to conduct member checks with original participants and the limited generalizability of the findings, as is consistent with qualitative studies in general. For patient data, translation is an automatic limitation, as the process could have affected data quality. For providers, the majority of interviews were conducted via telephone which could have affected overall interview length and quality; nonetheless, the team observed no substantive differences between the two data collection formats. The use of the model in other care settings may also incorporate additional variables. Interpreter perspectives were not included in the study due to a lack of access, but would be important for future studies. Finally, as with all novel theoretical frameworks, replication in future research studies is needed to further test its applicability for studying LAS implementation in healthcare settings.

### 4.2 Innovation

This study's primary innovation is the creation of a healthcare-specific theoretical framework that illustrates how to simultaneously integrate patient, clinician, and organizational factors into LS implementation studies that are linked to health outcomes. It offers flexibility to researchers in terms of the type of outcome chosen for study and can be aligned with organizational values and regulatory environments. The patient-centered side may help researchers to understand decision-making processes better as it highlights variables that may not have previously been considered important. Another innovation of this framework is that it could be used in tandem with existing implementation science frameworks. Merged frameworks may help to improve data capture associated with the complexity of delivering evidence-based interventions to patients in the presence of a language barrier.

Finally, the use of participants who spoke different languages, as well as monolingual and multilingual clinicians to evaluate the model using existing data, is innovative. Most studies focus on a single type of clinician or only on participants from one language group. Using data from the key actors involved in experiencing LS implementation helped provide a more complete picture than studying them separately.

### 4.3 Conclusion

Studying the challenges of implementing LAS to improve staff and patient experiences and outcomes would benefit from the use of LPT theories as an organizing framework for research studies. This study helped develop one such adaptation of LPT theory, specifically tailored to healthcare contexts. Through improved understanding of the implementation challenges, we have a better chance of addressing health outcome disparities associated with how language barriers affect health services delivery and the patient experience.

## CRediT authorship contribution statement

**Allison Squires:** Writing – review & editing, Writing – original draft, Validation, Supervision, Software, Resources, Project administration, Methodology, Investigation, Funding acquisition, Formal analysis, Data curation, Conceptualization. **Lauren Gerchow:** Writing – review & editing, Writing – original draft, Validation, Project administration, Formal analysis. **Chenjuan Ma:** Writing – review & editing, Writing – original draft, Formal analysis. **Eva Liang:** Writing – review & editing, Formal analysis, Data curation. **Sarah Miner:** Writing – review & editing, Writing – original draft, Project administration, Investigation, Formal analysis, Data curation.

## Consent for publication

The consent to participate in the study included a statement that the participant agreed to have their data used in publications and that anonymized quotes may be included in dissemination activities.

## Funding

This study was funded by the United States' Agency for Healthcare Research and Quality (AHRQ), R01HS023593. It provided support for all study implementation and dissemination activities.

## Declaration of competing interest

The authors declare no competing interests in relation to this study.

## Data Availability

The datasets used and/or analyzed during the current study are available from the corresponding author on reasonable request.
